# Supplementary material for: Effects of plant diversity on productivity strengthen over time due to trait-dependent shifts in species overyielding
Source: Nat Commun. 2024 Mar 7;15:2078. doi: 10.1038/s41467-024-46355-z (PMC10920907; doi:10.1038/s41467-024-46355-z)
Supplement: Supplementary file 1 — Supplementary Information [file 41467_2024_46355_MOESM1_ESM.pdf]

Supplementary Information

Effects of plant diversity on productivity strengthen over time due to trait-dependent shifts in species overyielding

Liting Zheng*, Kathryn E. Barry, Nathaly R. Guerrero-Ramírez, Dylan Craven, Peter B. Reich, Kris Verheyen, Michael Scherer-Lorenzen, Nico Eisenhauer, Nadia Barsoum, Jürgen Bauhus, Helge Bruelheide, Jeannine Cavender-Bares, Jiri Dolezal, Harald Auge, Marina V. Fagundes, Olga Ferlian, Sebastian Fiedler, David I. Forrester, Gislene Ganade, Tobias Gebauer, Josephine Haase, Peter Hajek, Andy Hector, Bruno Hérault, Dirk Hölscher, Kristin B. Hulvey, Bambang Irawan, Hervé Jactel, Julia Koricheva, Holger Kreft, Vojtech Lanta, Jan Leps, Simone Mereu, Christian Messier, Florencia Montagnini, Martin Mörsdorf, Sandra Müller, Bart Muys, Charles A. Nock, Alain Paquette, William C. Parker, John D. Parker, John A. Parrotta, Gustavo B. Paterno, Michael P. Perring, Daniel Piotto, H. Wayne Polley, Quentin Ponette, Catherine Potvin, Julius Quosh, Boris Rewald, Douglas L. Godbold, Jasper van Ruijven, Rachel J. Standish, Artur Stefanski, Leti Sundawati, Jon Urgoiti, Laura J. Williams, Brian J. Wilsey, Baiyu Yang, Li Zhang, Zhao Zhao, Yongchuan Yang, Hans Sandén, Anne Ebeling, Bernhard Schmid, Markus Fischer, Martyna M. Kotowska, Cecilia Palmborg, David Tilman, Enrong Yan*, Yann Hautier

Supplementary Table 1. Details of long-term grassland and forest biodiversity experiments included in the analysis.

Site	Country	Size of species pool	Species richness range	Number of yearly observations (Time span, years)
**Grassland**				
Agrodiversity[1]_BE	Belgium	4	1, 4	3 (1-3)
Agrodiversity_CA	Canada	4	1, 4	3 (1-3)
Agrodiversity_FR	France	4	1, 4	3 (1-3)
Agrodiversity_IS	Iceland	4	1, 4	3 (1-3)
Agrodiversity_IS	Iceland	4	1, 4	3 (1-3)
Agrodiversity_IE	Ireland	4	1, 4	3 (1-3)
Agrodiversity_IT	Italy	4	1, 4	3 (1-3)
Agrodiversity_LT_a	Lithuania	4	1, 4	3 (1-3)
Agrodiversity_LT_b	Lithuania	4	1, 4	3 (1-3)
Agrodiversity_LT_c	Lithuania	4	1, 4	3 (1-3)
Agrodiversity_NL	Netherlands	4	1, 4	3 (1-3)
Agrodiversity_NO_a	Norway	4	1, 4	3 (1-3)
Agrodiversity_NO_b	Norway	4	1, 4	3 (1-3)
Agrodiversity_NO_d	Norway	4	1, 4	3 (1-3)
Agrodiversity_PL	Poland	4	1, 4	3 (1-3)
Agrodiversity_SP_a	Spain	4	1, 4	3 (1-3)
Agrodiversity_SE_a	Sweden	4	1, 4	3 (1-3)
Agrodiversity_SE_b	Sweden	4	1, 4	3 (1-3)
Agrodiversity_SE_c	Sweden	4	1, 4	3 (1-3)
Agrodiversity_CH	Switzerland	4	1, 4	3 (1-3)
Agrodiversity_Wales_a	UK	4	1, 4	4 (1-4)
Agrodiversity_Wales_b	UK	4	1, 4	3 (1-3)
BigBio[2]	USA	18	1, 2, 4, 8, 16	16 (1-16)
BioCON[3]	USA	16	1, 4, 9, 16	14 (1-14)
BIODEPTH[4]_DE	Germany	31	1, 2, 4, 8, 16	3 (1-3)
BIODEPTH_GR	Greece	23	1, 2, 4, 8, 18	3 (1-3)
BIODEPTH_IE	Ireland	12	1, 2, 3, 4, 8	3 (1-3)
BIODEPTH_PT	Portugal	14	1, 2, 4, 8, 14	3 (1-3)
BIODEPTH_SW	Sweden	12	1, 2, 4, 8, 12	8 (1-8)
BIODEPTH_CH	Switzerland	48	1, 2, 4, 8	3 (1-3)
BIODEPTH_UK1	UK	12	1, 2, 4, 8, 12	3 (1-3)
BIODEPTH_UK2	UK	34	1, 2, 4, 8, 11	3 (1-3)

Hector_UK	UK	65	1, 3, 9, 27	3 (1-3)
Jena_Main_Mono[5,6]	Germany	60	1, 2, 4, 8, 16, 60	18 (1-18)
Lanta_drt[7]	Czech Republic	12	1, 3, 6	3 (1-3)
Lanta_nutr	Czech Republic	16	1, 2, 4, 8, 16	4 (1-4)
Texas_MEND[8]	USA	18	1, 9	3 (1-3)
Texas_Rich_Even[9]	USA	13	1, 2, 4, 8	10 (1-10)
Wagi_BioDiv[10]	Netherlands	8	1, 2, 4, 8	11 (1-11)
**Forest**				
BiodiversiTREE-SERC[11]	USA	16	1, 4, 12	2 (4-7)
BIOTREE-Species-K[12]	Germany	8	1, 2, 3, 4	2 (8-11)
BrazilDry (Brazil)	Brazil	16	1, 2, 4, 8, 16	2 (3-6)
B-Tree	Austria	4	1, 2, 4	3 (3-7)
Cann River[13]	Australia	2	1, 2	5 (3-11)
ClimateMatch-Kent	UK	4	1, 4	3 (3-9)
EFForTS-BEE[14]	Indonesia	6	1, 2, 3, 6	4 (3-5)
FDPutuo	China	8	1, 2, 4, 8	5 (3-5)
FORBIO[15]_Gedinne	Belgium	5	1, 2, 3, 4	5 (3-11)
FORBIO_Zedelgem	Belgium	5	1, 2, 3, 4	4 (3-8)
Hawaii[16]	USA	2	1, 2	9 (3 -11)
IDENT[17]_Cloquet	USA	12	1, 2, 6	9 (3-11)
IDENT_Freiburg	Germany	12	1, 2, 4, 6	6 (3-6)
IDENT_SSM	Canada	6	1, 2, 4, 6	5 (3-7)
IDENT_Macomer	Italy	12	1, 2, 4, 6	6 (3-8)
IDENT_Montreal	Canada	12	1, 2, 4, 12	9 (3-11)
Itatinga[18]	Brazil	2	1, 2	5 (3-6)
Kreinitz[19]	Germany	6	1, 2, 3, 5, 6	7 (3-11)
La selva[20] 1	Costa Rica	3	1, 3	5 (3 – 7)
La selva 2	Costa Rica	3	1, 3	3 (3 – 7)
La selva 3	Costa Rica	5	1, 5	4 (3 - 6)
MyDiv[21]	Germany	10	1, 2, 4	6 (3-6)
Ridgefield[22]	Australia	8	1, 2, 4, 8	4 (3-4)
Sardinilla[23]	Panama	6	1, 3, 6	9 (3-10)
Satakunta[24]	Finland	3	1, 2, 3	3 (3-11)
Toa Baja[25]	USA	2	1, 2	5 (3-7.5)

Supplementary Table 2. Linear mixed-effects models for effects of plant species richness (SR), year, and their interactions on community productivity and overyielding, as well as complementarity and selection effects in grasslands and forests. df, numerator degrees of freedom; ddf, denominator degrees of freedom; *F*, *F* ratios; *P*, *P* value of the significance test. Reported *P* values were calculated from one-sided *F*-test. Significant *P* (*P*<0.05) are denoted in bold.

	Grasslands				Forests				Forests			
	Biomass				Annual basal area increment				Accumulated basal area			
Community productivity												
Fixed	df	ddf	*F*	*P*	df	ddf	*F*	*P*	df	ddf	*F*	*P*
SR	1	1317	157.80	**<0.001**	1	1293	0.05	0.826	1	1348	13.61	**<0.001**
Year	1	5976	21.43	**<0.001**	1	3474	31.87	**<0.001**	1	4023	2229	**<0.001**
SR × Year	1	5976	7.96	**0.005**	1	3474	7.60	**0.006**	1	4023	12.59	**<0.001**
Random			Var.				Var.				Var.	
Plot_ID			2.15×10^{-4}				1.37				9.76×10^{-2}	
Site			3.19×10^{-1}				1.98×10^{1}				2.78×10^{-1}	
SR			9.23×10^{-3}				0.88×10^{-1}				3.10×10^{-2}	
Year			1.13×10^{-1}				7.13				4.03×10^{-2}	
Residual			7.70×10^{-2}				8.29				1.34×10^{-2}	
Year ρAR1			0.08				0.04				0.84	
Community overyielding												
Fixed	df	ddf	*F*	*P*	df	ddf	*F*	*P*	df	ddf	*F*	*P*
SR	1	716	78.09	**<0.001**	1	779	3.12	0.078	1	781	4.92	**0.027**
Year	1	3482	0.19	0.666	1	1934	0.84	0.361	1	2611	0.92	0.338
SR × Year	1	3482	39.97	**<0.001**	1	1934	12.57	**<0.001**	1	2611	19.37	**<0.001**
Random			Var.				Var.				Var.	
Plot_ID			1.52×10^{-5}				1.73×10^{-7}				4.26×10^{-2}	
Site			1.11×10^{-1}				5.71×10^{-2}				1.04×10^{-1}	
SR			2.66×10^{-3}				1.49×10^{-2}				8.73×10^{-3}	
Year			2.38×10^{-2}				4.68×10^{-2}				1.77×10^{-2}	
Residual			2.49×10^{-1}				1.64×10^{-1}				3.15×10^{-2}	
Year ρAR1			0.21				0.03				0.67	
Complementarity effect												
Fixed	df	ddf	*F*	*P*	df	ddf	*F*	*P*	df	ddf	*F*	*P*
SR	1	700	9.15	**0.002**	1	779	0.10	0.749	1	781	9.82	**0.002**
Year	1	3223	1.49	0.222	1	1930	0.95	0.330	1	2611	0.13	0.723
SR × Year	1	3223	27.97	**<0.001**	1	1930	1.12	0.287	1	2611	11.72	**<0.001**
Random			Var.				Var.				Var.	

Plot_ID			2.24×10^{-1}				4.66×10^{-3}				1.72×10^{-1}	
Site			3.47×10^{3}				1.56×10^{1}				3.09×10^{1}	
SR			6.47×10^{2}				3.76×10^{-2}				1.68×10^{-1}	
Year			3.40×10^{-1}				8.26				3.11×10^{1}	
Residual			2.60×10^{4}				8.82				8.57	
Year ρAR1			0.21				0.20				0.43	
Selection effect												
Fixed	df	ddf	*F*	*P*	df	ddf	*F*	*P*	df	ddf	*F*	*P*
SR	1	700	0.03	0.856	1	779	0.83	0.363	1	781	1.55	0.213
Year	1	3223	0.08	0.775	1	1930	0.15	0.701	1	2611	0.13	0.716
SR × Year	1	3223	5.22	**0.022**	1	1930	1.67	0.198	1	2611	3.44	0.062
Random			Var.				Var.				Var.	
Plot_ID			3.46×10^{2}				1.78×10^{-2}				4.94×10^{-2}	
Site			4.25×10^{3}				8.80				3.15×10^{1}	
SR			6.61×10^{-1}				6.36×10^{-2}				2.10×10^{-2}	
Year			2.88×10^{3}				5.78				2.51×10^{1}	
Residual			1.45×10^{4}				3.81				3.24	
Year ρAR1			0.20				-0.06				0.62	

* Random factors include random intercept for PlotID (Plot_ID), random intercept for each site (Site), random slopes for species richness (SR) and year (Year) per site. Year ρAR1 indicates the temporal autocorrelation of residuals across years.

Supplementary Table 3. The summary of principal component analyses of economics traits of the grassland species and forest tree species based on imputed (missing trait values were imputed) and complete (complete measured traits) datasets. Displayed is the Eigenvalue as well as the proportion of variance explained by each principal component (PC) and the loadings of the economics traits. SLA: specific leaf area, LDMC: leaf dry matter content, LNC: leaf nitrogen content, SRL: specific root length, RNC: root nitrogen content, RTD: root tissue density.

	Regression models	PC1	PC2	Trait data description (n. measured / n. imputed)
	Eigenvalue	2.12	1.45	
	Variance	0.37	0.24	
**Grassland species**	SLA	1.70	0.32	(165 / 1)
**(Imputed)**	LDMC	-1.45	-0.36	(148 / 18)
n=166	LNC	0.61	1.82	(152 / 14)
	SRL	1.80	-0.55	(136 / 30)
	RNC	-0.26	1.93	(127 / 39)
	RTD	-1.71	-0.18	(120 / 46)
	Eigenvalue	1.87	1.55	
	Variance	0.31	0.26	
**Forest tree species**	SLA	1.72	0.68	(133 / 1)
**(Imputed)**	LDMC	-0.51	-1.38	(98 / 36)
n=134	LNC	1.93	-0.01	(121 / 13)
	SRL	0.17	1.34	(97 / 37)
	RNC	1.33	-0.01	(67 / 67)
	RTD	0.23	-1.77	(81 / 53)
	Eigenvalue	2.04	1.48	
	Variance	0.34	0.25	
**Grassland species**	SLA	1.47	0.15	(94 / 0)
**(Complete)**	LDMC	-1.20	-0.37	(94 / 0)
n=94	LNC	0.46	1.59	(94 / 0)
	SRL	1.47	-0.65	(94 / 0)
	RNC	-0.18	1.64	(94 / 0)
	RTD	-1.43	-0.17	(94 / 0)
	Eigenvalue	1.96	1.62	
	Variance	0.33	0.27	
**Forest tree species**	SLA	1.14	0.92	(42 / 0)
**(Complete)**	LDMC	-0.23	-1.24	(42 / 0)
n=42	LNC	1.36	-0.36	(42 / 0)
	SRL	0.59	0.50	(42 / 0)
	RNC	1.25	-0.32	(42 / 0)
	RTD	0.01	-1.16	(42 / 0)

* Numbers of trait data descriptions for each trait indicate the number of species with measured and imputed values, respectively.

Supplementary Table 4. Variance components of random terms in linear mixed-effects models for effects of plant economics traits (represented by trait PC1 and PC2), species richness (SR), year, and their interactions on species species log response ratio of aboveground biomass in grasslands and annual basal area increment and accumulated basal area in forests.

	Grasslands	Forests	
	Biomass	Annual basal area increment	Accumulated basal area
**Fixed terms: PC1 + SR + Year + PC1 × SR + PC1 × Year + SR × Year**			
Random	Var.	Var.	Var.
SP_PlotID	1.62×10^{-2}	1.80×10^{-2}	8.87×10^{-2}
Site	3.62×10^{-2}	3.17×10^{-2}	1.31×10^{-1}
PC1	8.97×10^{-2}	1.72×10^{-2}	1.27×10^{-2}
Year	1.42×10^{-1}	1.34×10^{-1}	2.76×10^{-1}
SR	9.44×10^{-2}	6.08×10^{-2}	2.09×10^{-2}
Residual	1.86	4.90×10^{-1}	6.75×10^{-2}
Temporal autocorrelation			
Year ρAR1	0.45	0.03	0.63
R^2	$R^2_m = 0.02$, $R^2_c = 0.40$	$R^2_m = 0.01$, $R^2_c = 0.54$	$R^2_m = 0.01$, $R^2_c = 0.87$
**Fixed terms: PC2 + SR + Year + PC2 × SR + PC2 × Year + SR × Year**			
Random	Var.	Var.	Var.
SP_PlotID	1.38×10^{-2}	9.46×10^{-3}	2.24×10^{-3}
Site	1.53×10^{-2}	1.27×10^{-3}	2.36×10^{-1}
PC2	1.44×10^{-2}	6.56×10^{-3}	4.91×10^{-2}
Year	1.49×10^{-1}	1.49×10^{-1}	2.46×10^{-1}
SR	9.74×10^{-2}	8.44×10^{-2}	3.39×10^{-2}
Residual	1.84	4.80×10^{-1}	7.35×10^{-2}
Temporal autocorrelation			
Year ρAR1	0.45	0.03	0.67
R^2	$R^2_m = 0.02$, $R^2_c = 0.41$	$R^2_m = 0.02$, $R^2_c = 0.53$	$R^2_m = 0.02$, $R^2_c = 0.86$

* Random factors include a random intercept for each species at each unique plot (SP_PlotID), a random intercept for each site (Site), and random slopes for species richness (SR), species functional traits (PC1/PC2), and year (Year) per site. Year ρAR1 indicates the temporal autocorrelation of residuals across years. R^2_m and R^2_c represent the amounts of explained variances by fixed variables and all fixed and random variables, respectively.

Supplementary Table 5. Linear mixed-effects models for effects of plant economic traits, species richness (SR), experimental year (Year), and their interactions on species log response ratio of aboveground biomass in grasslands. df, numerator degrees of freedom; ddf, denominator degrees of freedom; *F*, *F* ratios; *P*, *P* value of the significance test. Reported *P* values were calculated from one-sided *F*-test. Significant *P* values (P<0.05) are denoted in bold.

Predictors	df	ddf	*F*	*P*
Specific leaf area	1	3536	0.09	0.764
SR	1	3536	9.88	**0.002**
Year	1	14717	9.81	**0.002**
Specific leaf area × SR	1	3536	8.46	**0.004**
Specific leaf area × Year	1	14717	12.94	**<0.001**
SR × Year	1	14717	26.88	**<0.001**
Specific root length	1	3536	3.34	0.068
SR	1	3536	0.93	0.335
Year	1	14717	6.01	**0.014**
Specific root length × SR	1	3536	0.41	0.524
Specific root length × Year	1	14717	8.54	**0.003**
SR × Year	1	14717	23.84	**<0.001**
Leaf dry matter content	1	3536	1.91	0.167
SR	1	3536	1.63	0.202
Year	1	14717	1.56	0.212
Leaf dry matter content × SR	1	3536	2.52	0.113
Leaf dry matter content× Year	1	14717	2.49	0.115
SR × Year	1	14717	30.66	**<0.001**
Root tissue density	1	3536	2.15	0.142
SR	1	3536	0.23	0.630
Year	1	14717	2.09	0.148
Root tissue density × SR	1	3536	0.09	0.767
Root tissue density × Year	1	14717	6.22	**0.013**
SR × Year	1	14717	24.58	**<0.001**
Leaf nitrogen content	1	3536	2.81	0.094
SR	1	3536	4.27	**0.039**
Year	1	14717	53.79	**<0.001**
Leaf nitrogen content × SR	1	3536	3.39	0.065
Leaf nitrogen content × Year	1	14717	62.63	**<0.001**
SR × Year	1	14717	21.85	**<0.001**
Root nitrogen content	1	3536	3.00	0.083
SR	1	3536	9.36	**0.002**
Year	1	14717	0.55	0.459
Root nitrogen content × SR	1	3536	6.32	**0.012**
Root nitrogen content × Year	1	14717	1.69	0.194
SR × Year	1	14717	28.61	**<0.001**

Supplementary Table 6. Linear mixed-effects models for effects of plant economic traits, species richness (SR), experimental year, and their interactions on species species log response ratio of annual basal area increment and accumulated total basal area in forests. df, numerator degrees of freedom; ddf, denominator degrees of freedom; *F*, *F* ratios; *P*, *P* value of the significance test (calculated from one-sided *F*-test). Significant *P* (*P*<0.05) are denoted in bold. Abbreviations of traits are given in Table S1.

	Annual basal area increment				Accumulated basal area			
Predictors	Df	ddf	*F*	*P*	df	ddf	*F*	*P*
Specific leaf area	1	2430	3.06	0.081	1	2460	0.26	0.610
SR	1	2430	1.38	0.241	1	2460	0.11	0.737
Year	1	6028	10.13	**0.001**	1	7783	0.07	0.786
Specific leaf area × SR	1	2430	0.12	0.731	1	2460	0.99	0.319
Specific leaf area × Year	1	6028	18.89	**<0.001**	1	7783	1.36	0.243
SR × Year	1	6028	3.52	0.060	1	7783	0.20	0.658
Leaf nitrogen content	1	2430	0.62	0.431	1	2460	1.77	0.184
SR	1	2430	9.45	**0.002**	1	2460	1.79	0.182
Year	1	6028	6.01	**0.014**	1	7783	10.26	**0.001**
Leaf nitrogen content × SR	1	2430	7.84	**0.005**	1	2460	1.24	0.265
Leaf nitrogen content × Year	1	6028	7.56	**0.006**	1	7783	8.60	**0.003**
SR × Year	1	6028	1.84	0.175	1	7783	0.19	0.663
Root nitrogen content	1	2430	5.26	**0.022**	1	2460	0.69	0.406
SR	1	2430	10.05	**0.002**	1	2460	1.73	0.188
Year	1	6028	11.29	**0.001**	1	7783	14.77	**<0.001**
Root nitrogen content × SR	1	2430	8.08	**0.004**	1	2460	1.11	0.293
Root nitrogen content × Year	1	6028	10.92	**0.001**	1	7783	12.67	**<0.001**
SR × Year	1	6028	2.47	0.116	1	7783	0.28	0.597
Leaf dry matter content	1	2430	2.40	0.121	1	2460	0.01	0.948
SR	1	2430	1.81	0.179	1	2460	1.79	0.181
Year	1	6028	13.33	**<0.001**	1	7783	1.37	0.243
Leaf dry matter content × SR	1	2430	1.42	0.234	1	2460	1.51	0.219
Leaf dry matter content ×Year	1	6028	14.40	**<0.001**	1	7783	1.88	0.171
SR × Year	1	6028	1.84	0.175	1	7783	0.22	0.640
Specific root length	1	2430	0.12	0.727	1	2460	3.23	0.072
SR	1	2430	8.65	**0.003**	1	2460	1.30	0.254
Year	1	6028	0.79	0.374	1	7783	33.22	**<0.001**
Specific root length × SR	1	2430	6.63	**0.010**	1	2460	0.47	0.495
Specific root length × Year	1	6028	0.33	0.568	1	7783	34.11	**<0.001**
SR × Year	1	6028	2.18	0.140	1	7783	0.24	0.626
Root tissue density	1	2430	6.31	0.012	1	2460	0.05	0.825
SR	1	2430	1.42	0.233	1	2460	0.95	0.330
Year	1	6028	14.23	**<0.001**	1	7783	7.29	**0.007**
Root tissue density × SR	1	2430	0.08	0.779	1	2460	0.01	0.960
Root tissue density × Year	1	6028	17.61	**<0.001**	1	7783	5.81	**0.016**
SR × Year	1	6028	2.36	0.124	1	7783	0.23	0.633

Supplementary Table 7. Comparison of models to test the effects of plant economic traits (represented by traits PC1 and traits PC2), species richness (SR), year, and their interactions on species log response ratio of aboveground biomass in grasslands and annual basal area increment and accumulated basal area in forests with or without with or without climate covariates. MAT, mean annual temperature; MAP, mean annual precipitation; SPEI, standardized precipitation-evapotranspiration index; DF, numerator degrees of freedom and denominator degrees of freedom; *F*, *F* ratios; *P*, *P* value of the significance test. Reported *P* values were calculated from one-sided *F*-test. Significant *P* (*P*<0.05) are denoted in bold.

	Models with climate covariate								
	MAT			*MAP*			*SPEI*		
	Df	*F*	*P*	df	*F*	*P*	df	*F*	*P*
**Grasslands**	Responses: Species species log response ratio of biomass								
Traits PC1	1,3536	0.70	0.403	1,3536	0.53	0.465	1,3536	0.46	0.496
SR	1,3536	2.31	0.129	1,3563	2.95	0.086	1,3536	2.59	0.108
Year	1,14716	0.34	0.561	1,14716	0.27	0.601	1,14716	0.22	0.638
Traits PC1 × SR	1,3561	2.30	0.130	1,3536	2.65	0.104	1,3536	2.52	0.113
Traits PC1 × Year	1,14716	13.56	**0.001**	1,14716	13.41	**<0.001**	1,14716	12.57	**<0.001**
SR × Year	1,14716	25.99	**<0.001**	1,14716	25.54	**<0.001**	1,14716	25.68	**<0.001**
Climate covariate	1,14716	1.17	0.280	1,14716	1.27	0.260	1,14716	2.08	0.149
AIC	63415			63427			63413		
AIC of Model without climate covariate				62083					
Traits PC2	1,3536	0.13	0.723	1,3536	0.05	0.820	1,3536	0.07	0.790
SR	1,3536	6.42	**0.011**	1,3536	6.90	**0.009**	1,3536	6.32	**0.012**
Year	1,14716	1.36	0.243	1,14716	1.44	0.229	1,14716	1.11	0.293
Traits PC2 × SR	1,3561	5.98	**0.014**	1,3536	5.07	**0.024**	1,3536	5.55	**0.019**
Traits PC2 × Year	1,14716	20.26	**<0.001**	1,14716	20.28	**<0.001**	1,14716	19.40	**<0.001**
SR × Year	1,14716	33.40	**<0.001**	1,14716	34.96	**<0.001**	1,14716	34.26	**<0.001**
Climate covariate	1,14716	0.01	0.950	1,14716	1.94	0.164	1,14716	3.92	**0.048**
AIC	63458			63469			63452		
AIC of Model without climate covariate				62151					
**Forests**	Responses: Species species log response ratio of annual basal area increment								
Traits PC1	1, 2430	0.82	0.365	1, 2430	0.96	0.327	1, 2430	0.91	0.340
SR	1,2430	2.46	0.117	1,2430	2.54	0.111	1,2430	2.13	0.144
Year	1, 6027	0.19	0.661	1, 6027	0.13	0.717	1, 6027	0.15	0.698
Traits PC1 × SR	1, 2430	1.17	0.280	1, 2430	1.09	0.296	1, 2430	1.37	0.242
Traits PC1 × Year	1, 6027	6.18	**0.013**	1, 6027	5.72	**0.017**	1, 6027	6.06	**0.013**
SR × Year	1, 6027	2.23	0.135	1, 6027	2.12	0.146	1, 6027	1.93	0.165
Climate covariate	1, 6027	0.06	0.806	1, 6027	2.78	0.095	1, 6027	1.98	0.159
AIC	21710			21720			21705		
AIC of Model without climate covariate				21546					

Traits PC2	1, 2430	4.36	0.037	1, 2430	3.61	0.057	1, 2430	4.15	0.042
SR	1,2430	1.49	0.221	1,2430	1.70	0.192	1,2427	1.29	0.257
Year	1, 6027	0.05	0.829	1, 6027	0.03	0.865	1, 6027	0.03	0.873
Traits PC2 × SR	1, 2430	0.13	0.720	1, 2430	0.04	0.841	1, 2430	0.11	0.742
Traits PC2 × Year	1, 6027	18.68	**<0.001**	1, 6027	17.58	**<0.001**	1, 6027	18.87	**<0.001**
SR × Year	1, 6027	1.30	0.254	1, 6027	1.32	0.249	1, 6027	1.07	0.300
Climate covariate	1, 6027	0.01	0.921	1, 6027	2.61	0.106	1, 6027	0.99	0.318
AIC	21717			21726			21713		
AIC of Model without climate covariate				21570					
**Forests**	Responses: Species log response ratio of accumulated basal area								
Traits PC1	1, 2460	1.55	0.213	1, 2460	1.56	0.212	1, 2460	1.59	0.207
SR	1, 2460	1.89	0.168	1, 2460	2.85	0.091	1, 2460	2.84	0.092
Year	1, 7782	2.38	0.122	1, 7782	1.96	0.161	1, 7782	1.81	0.179
Traits PC1 × SR	1, 2460	0.50	0.480	1, 2460	0.17	0.676	1, 2460	3.02	0.569
Traits PC1 × Year	1, 7782	4.62	**0.032**	1, 7782	4.65	**0.031**	1, 7782	4.74	**0.029**
SR × Year	1, 7782	0.26	0.609	1, 7782	0.17	0.680	1, 7782	0.22	0.638
Climate covariate	1, 7782	10.33	**0.001**	1, 7782	5.50	**0.019**	1, 7782	3.02	0.082
AIC	6582			6594			6592		
AIC of Model without climate covariate		5803							
**Forests**	Responses: Species log response ratio of accumulated basal area								
Traits PC2	1, 2460	2.41	0.121	1, 2460	1.11	0.291	1, 2460	2.27	0.132
SR	1, 2460	1.88	0.170	1, 2460	3.05	0.081	1, 2460	2.82	0.093
Year	1, 7782	2.48	0.115	1, 7782	2.10	0.148	1, 7782	1.98	0.160
Traits PC2 × SR	1, 2460	0.01	0.927	1, 2460	0.03	0.869	1, 2460	0.03	0.864
Traits PC2 × Year	1, 7782	24.48	**<0.001**	1, 7782	23.54	**<0.001**	1, 7782	24.70	**<0.001**
SR × Year	1, 7782	0.29	0.587	1, 7782	0.20	0.655	1, 7782	0.32	0.573
Climate covariate	1, 7782	8.88	**0.003**	1, 7782	5.17	**0.023**	1, 7782	3.75	0.053
AIC	6567			6598			6587		
AIC of Model without climate covariate		5829							

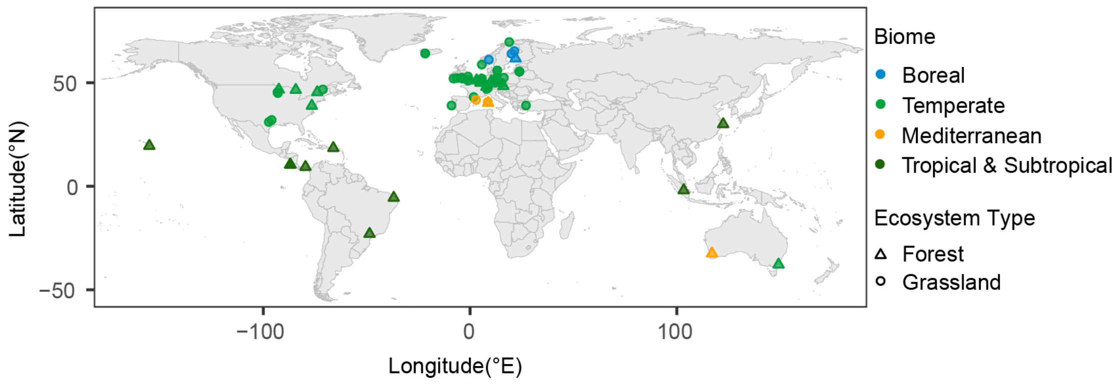

Supplementary Figure 1. Map of locations of the grassland and forest experiments included in the study. The 39 grassland experiments are in temperate (n=34), Mediterranean (n=2), and boreal (n=3) biomes. The 26 forest experiments are in temperate (n=13), tropical and subtropical (n=10), mediterranean (n=2), and boreal (n=1) biomes. The worldmap was produced using the R package '*tmap*'.

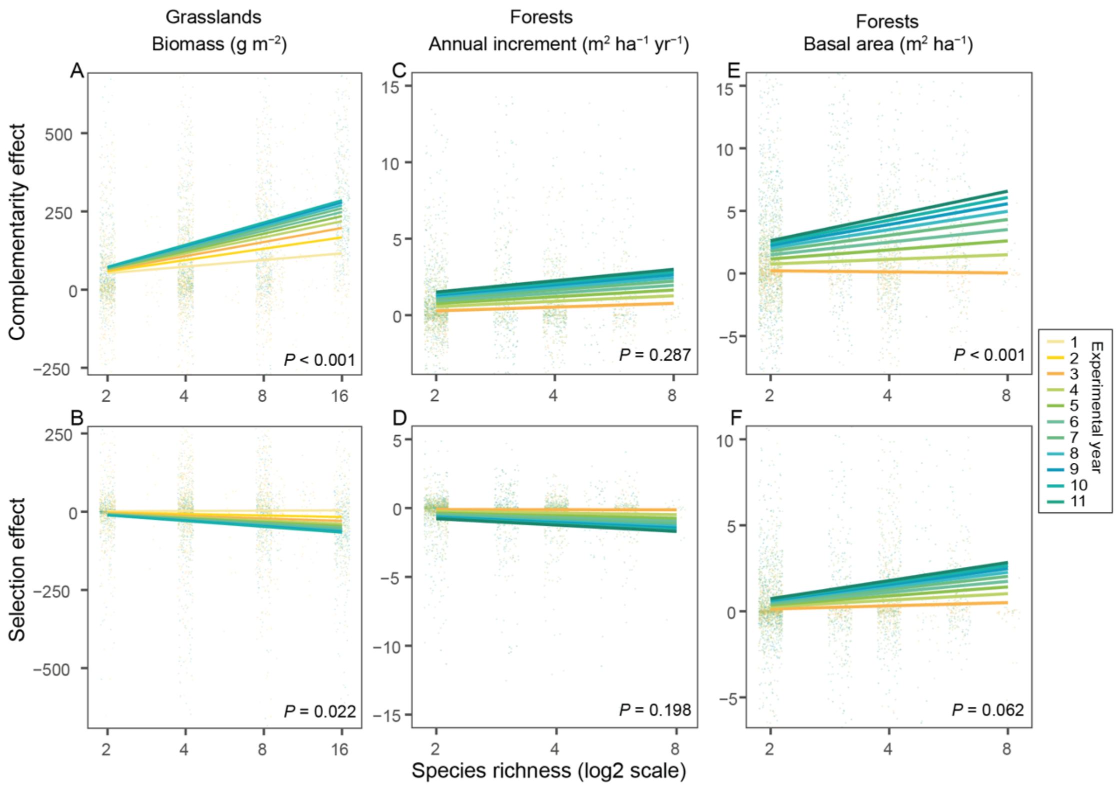

Supplementary Figure 2. The relationships of complementarity effect and selection effect with plant species richness over time in grassland and forest ecosystems. A-B, The effects of species richness on complementarity (A) and selection effects (B) in terms of aboveground biomass in grasslands. C-F, The effects of species richness on complementarity and selection effects in terms of annual basal area increment (C-D) and accumulated total basal area (E-F) in forests. Points are community-level values for each plot in the respective year. Lines are mixed effect model fits across all experiments. *P* values are derived from the significance tests (one-sided *F*-test) of the interaction term for species richness and experimental years in the ANOVA test on linear mixed models. Refer to Supplementary Table 3 for detailed statistical analyses. To enhance resolution comparing model fit lines, y-axis legends were used that mask the most extreme individual values (i.e., 5% of total values are not visible).

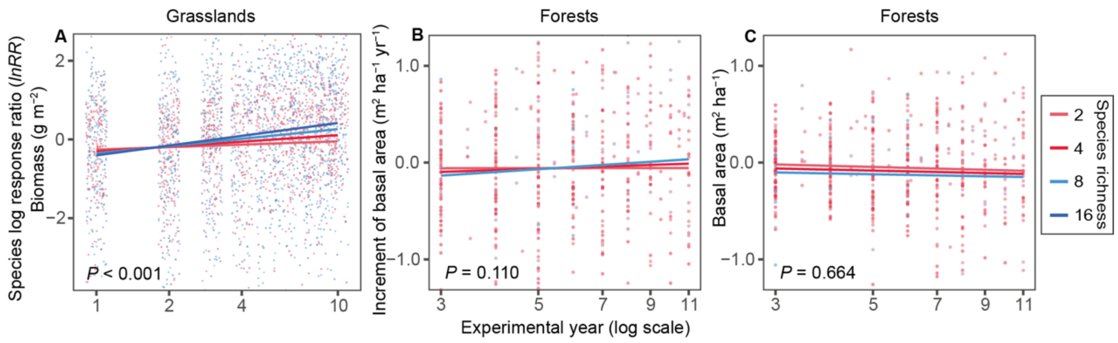

Supplementary Figure 3. The species overyielding (or underyielding) in mixtures in relation to plant species richness across experimental years in grassland and forest experimental ecosystems. Positive species log response ratio (*lnRR)* indicates overyielding and negative value indicates underyielding. A, The relationship between species *lnRR* of above-ground biomass with species richness in grasslands. B-C, The relationship between species lnRR of annual basal area increment (B) and accumulated total basal area (C) with species richness in forests. *P* values are derived from the significance tests (one-sided *F*-test) of the interaction term for species richness and experimental years in the ANOVA test on linear mixed models (based on Traits PC1, refer to Table 1 for more details).

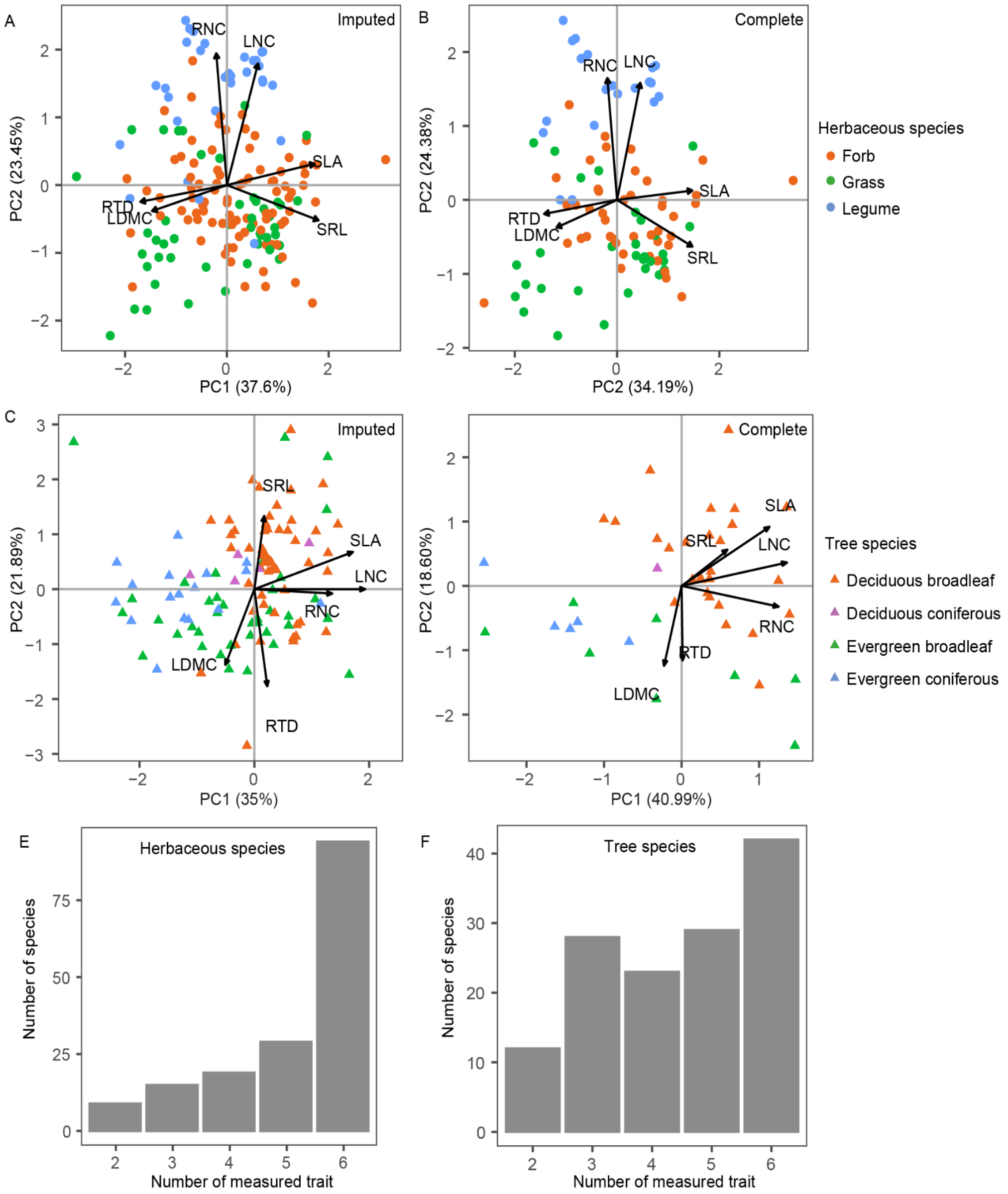

Supplementary Figure 4. Principal component analyses (PCA) of six economics traits of the herbaceous species in grasslands and tree species in forests. A, PCA of imputed traits for the herbaceous species (n=166). B, PCA of complete measured traits for the herbaceous species (n=94). C, PCA of imputed traits of the tree species (n=134). D, PCA of complete measured traits of the tree species (n=42). E-F, The distribution of available trait number for herbaceous species (median=6) and tree species (median=5) used for imputation. Abbreviations of traits are given in Table S1.

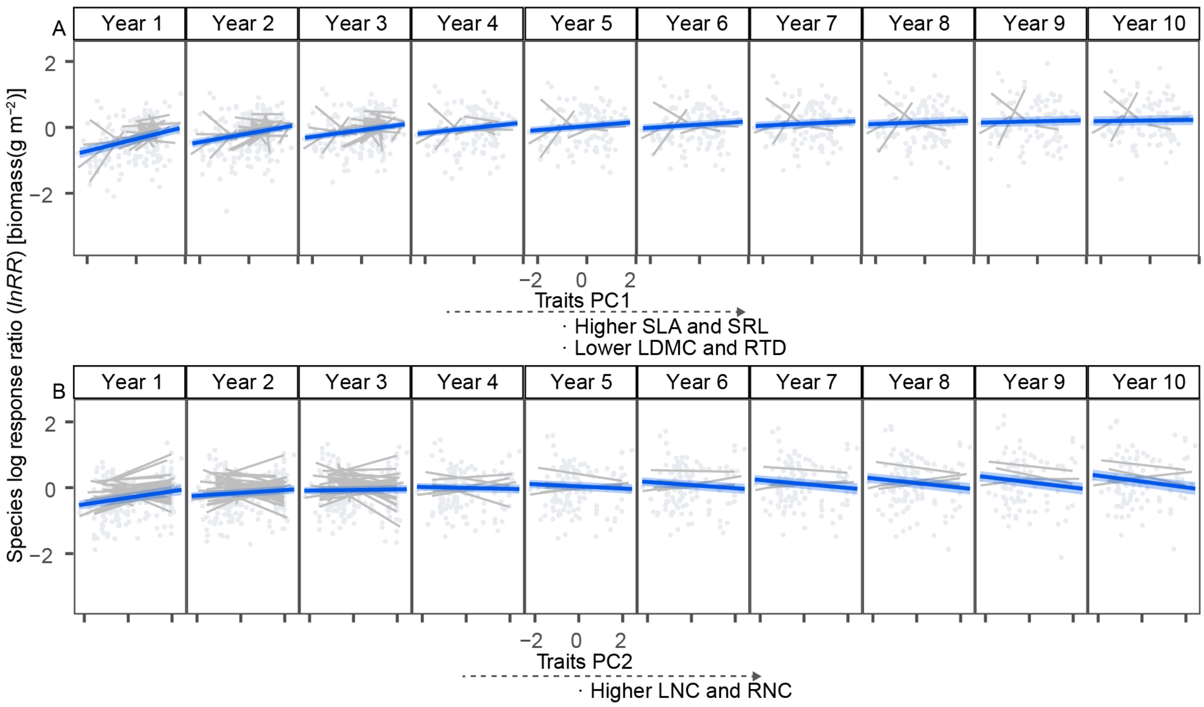

Supplementary Figure 5. Species log response ratio in mixed-species communities in relation to plant economics traits (A: Traits PC1; B: Traits PC2) across all experiments (blue thick lines with bands indicating 95% confidence intervals) and within each experiment (grey thin lines) over experimental years in grassland experimental ecosystems. Points are the mean log response for each combination of species and year in each experiment.

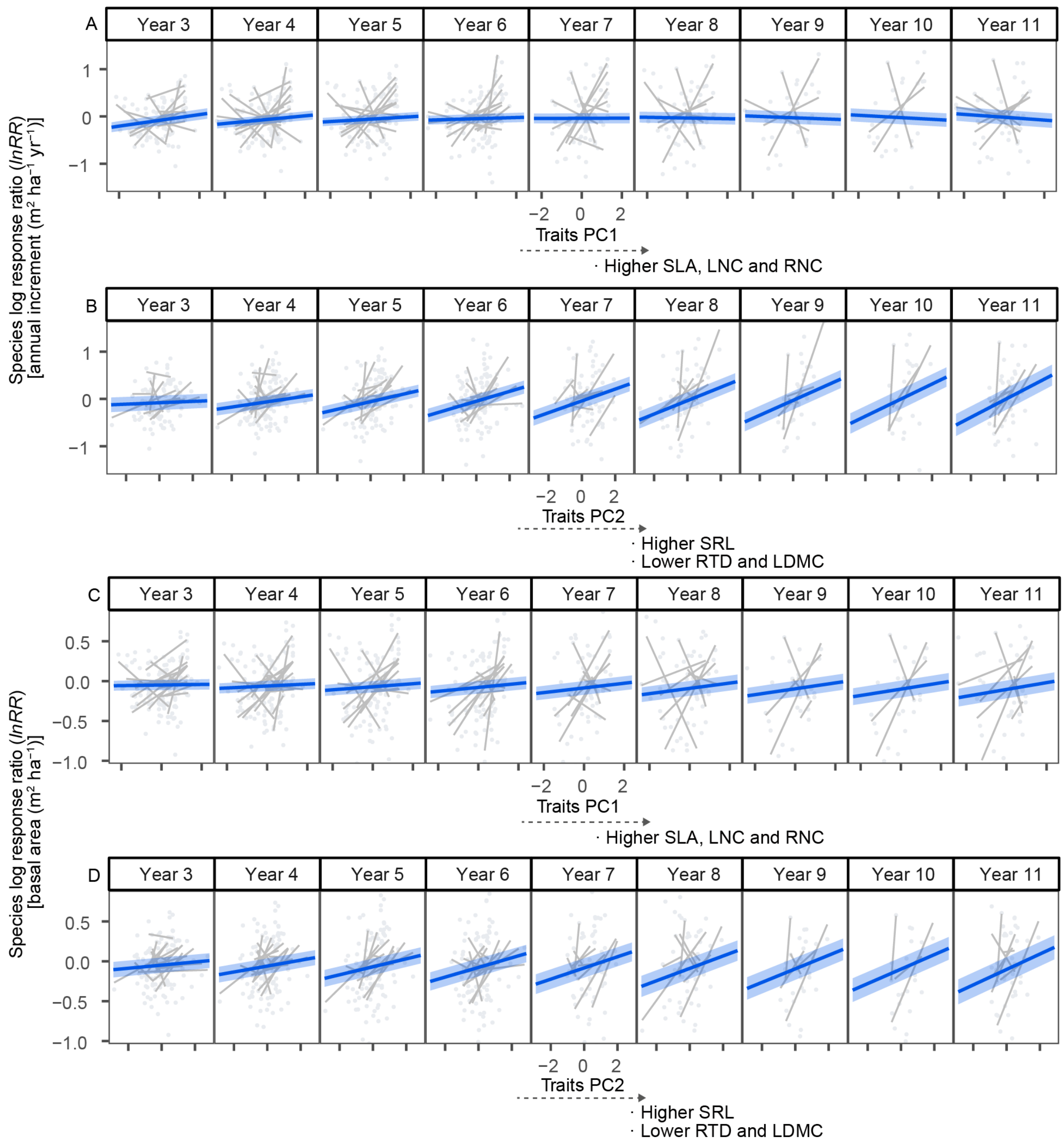

Supplementary Figure 6. Species log response ratio in mixed-species communities in relation to plant economics traits across all experiments (blue thick lines with bands indicating 95% confidence intervals) and within each experiment (grey thin lines) over experimental years in forest experimental ecosystems. A-B, The relationship between species *lnRR* of annual basal area increment with plant economics traits. C-D, The relationship between species *lnRR* of accumulated basal area with plant economics traits. Points are the mean species response ratio for each combination of species and year in each experiment.

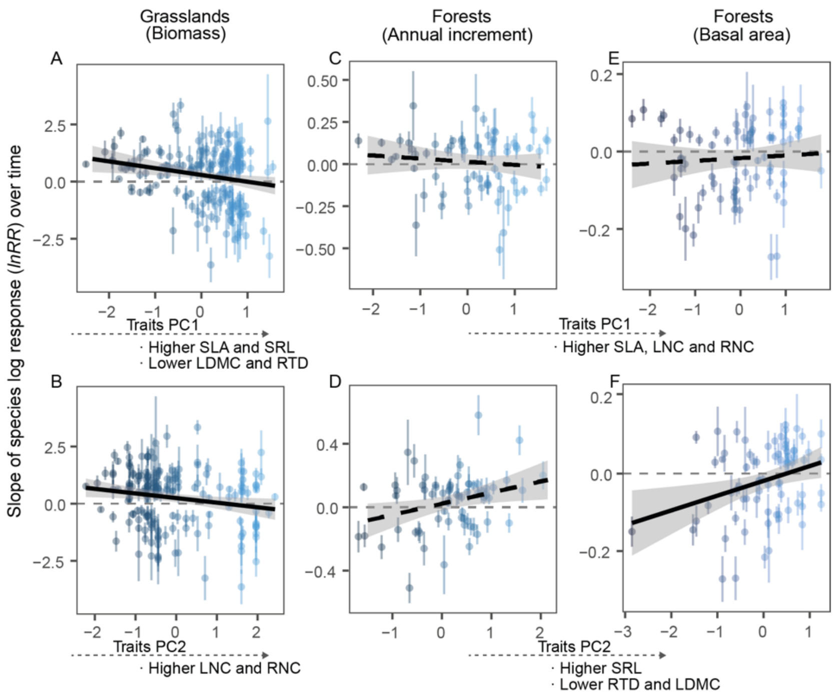

Supplementary Figure 7. Slopes of species log response ratio (lnRR) over time in relation to plant economics traits in grassland and forest ecosystems. Circles represent the slopes of species *lnRR* over time of each species, and error bars are 95% credible intervals on the mean. Only the significant slopes are shown. Positive slope indicates species *lnRR* increases over time, whereas negative slope indicates *lnRR* decreases over time. A-B, In grasslands, the slopes of species *lnRR* over time based on biomass decreased with species traits PC1 ($P = 0.009$) and PC2 ($P = 0.018$). C-F, In forests, the slopes of species *lnRR* over time based on annual basal area increment decreased with species traits PC1 ($P = 0.645$) but increased with PC2 ($P = 0.535$), whereas the slopes of species *lnRR* based on accumulated total basal area over time weakly increased with species traits PC1 ($P = 0.600$) and PC2 ($P = 0.022$). *P* values are derived from the two-sided *t* test.

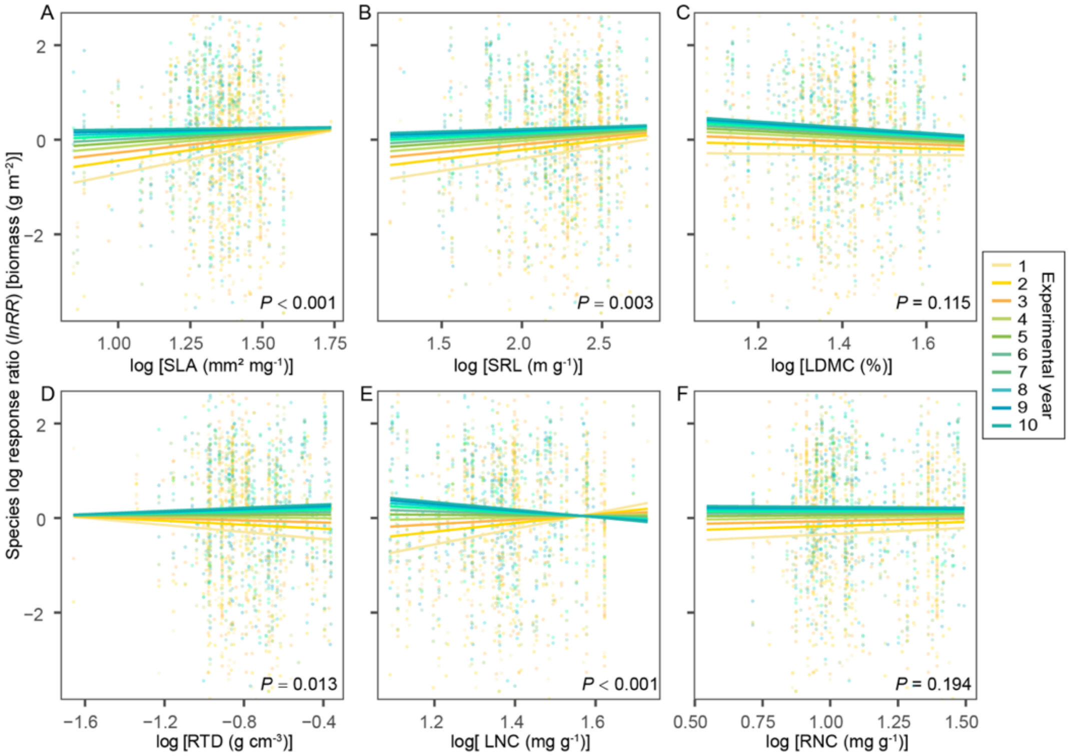

Supplementary Figure 8. Species overyielding (or underyielding) of aboveground biomass in mixed-species communities in relation to plant economics traits (including gap-filling values) across experimental years in grasslands (n=39). Positive species log response ratio (*lnRR)* indicates overyielding and negative value indicates underyielding. Points represent the *lnRR* for each species and year in each experiment. *P* values are derived from the significance tests (one-sided *F*-test) of the interaction term for plant economics traits and experimental years in the ANOVA test on linear mixed models.

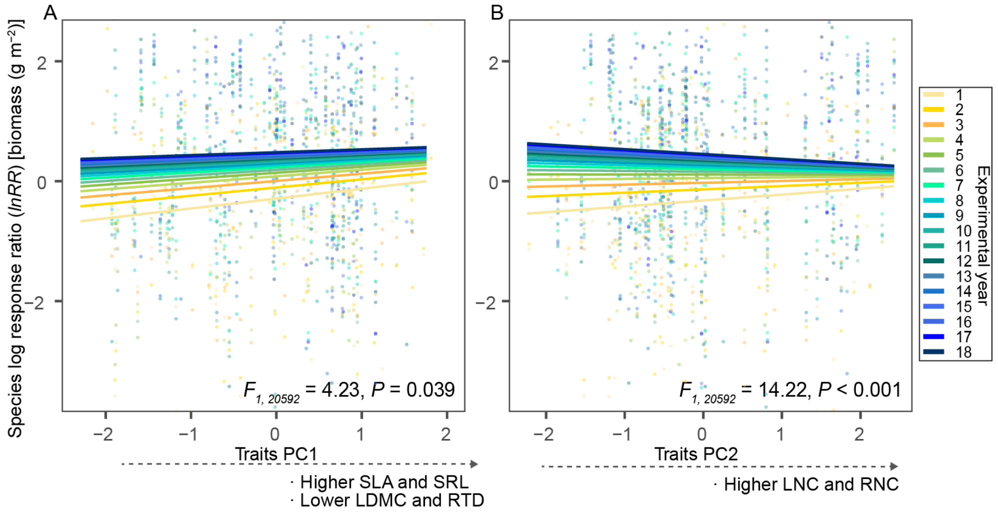

Supplementary Figure 9. Species overyielding (or underyielding) of aboveground biomass in mixtures in relation to plant economics traits (including gap-filling values) across experimental years in grassland experimental ecosystems across 18 years. Positive species log response ratio (*lnRR)* indicates overyielding and negative value indicates underyielding. Points represent the *lnRR* for each species and year in each experiment. *P* values are derived from the significance tests (one-sided *F*-test) of the interaction term for plant economics traits and experimental years in the ANOVA test on linear mixed models.

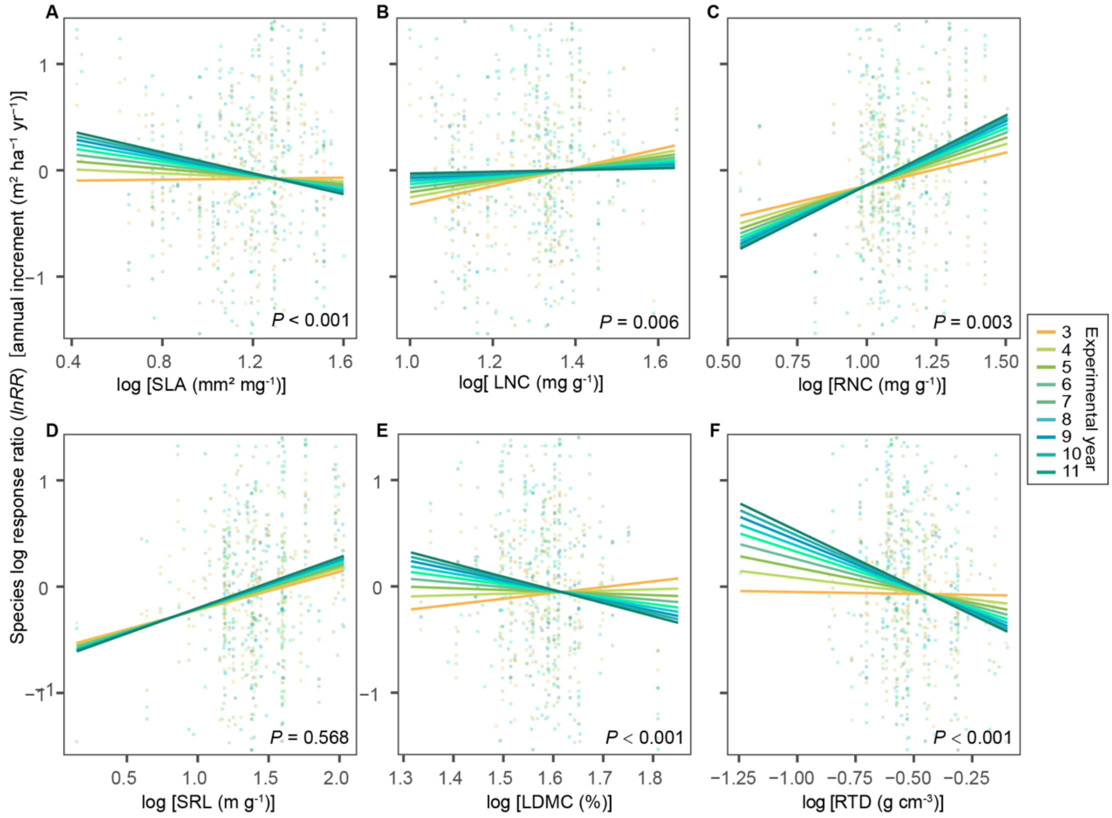

Supplementary Figure 10. Species overyielding (or underyielding) of annual increment of the basal area in relation to plant economics traits (including gap-filling values) across experimental years in forests (n= 26). Positive species log response ratio (*lnRR)* indicates overyielding and negative value indicates underyielding. Points represent the *lnRR* for each species and year in each experiment. *P* values are derived from the significance tests (one-sided *F*-test) of the interaction term for plant economics traits and experimental years in the ANOVA test on linear mixed models. Abbreviations of traits are given in Table S1.

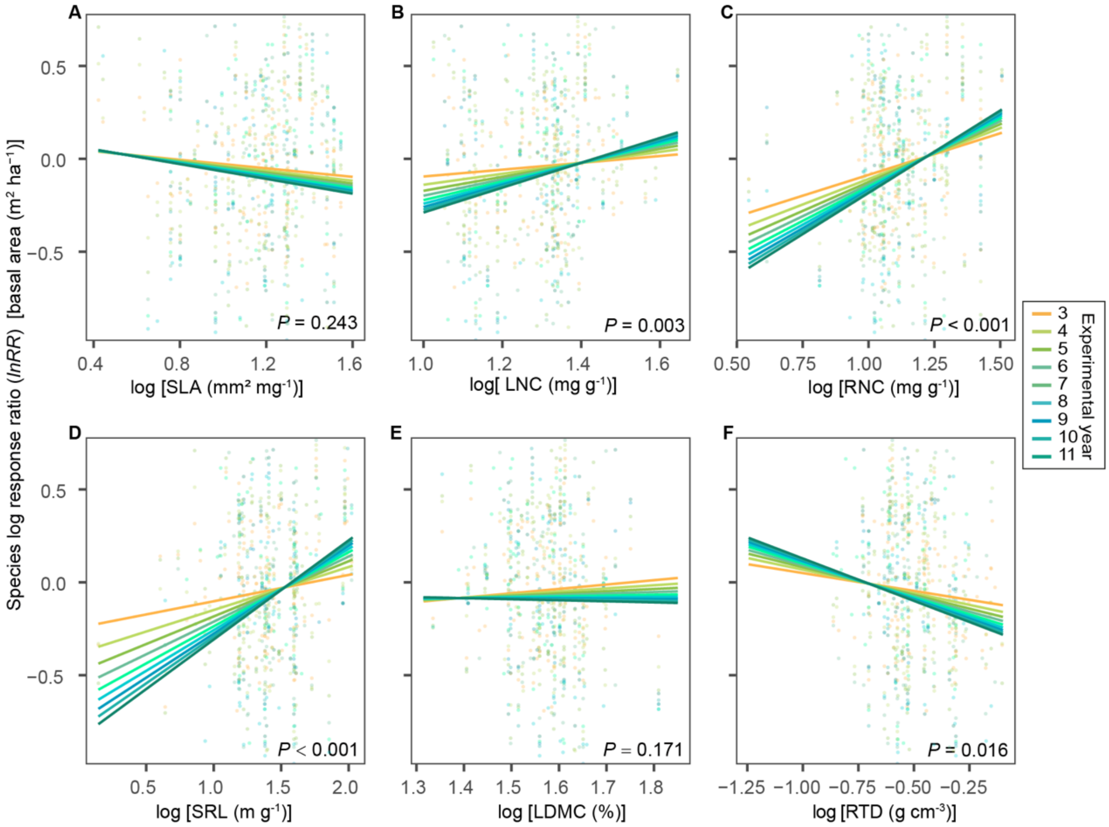

Supplementary Figure 11. Species overyielding (or underyielding) of accumulated total basal area in relation to plant economics traits (including gap-filling values) across experimental years in forests (n=26). Positive species log response ratio (*lnRR)* indicates overyielding and negative value indicates underyielding. Points represent the *lnRR* for each species and year in each experiment. *P* values are derived from the significance tests (one-sided *F*-test) of the interaction term for plant economics traits and experimental years in the ANOVA test on linear mixed models.

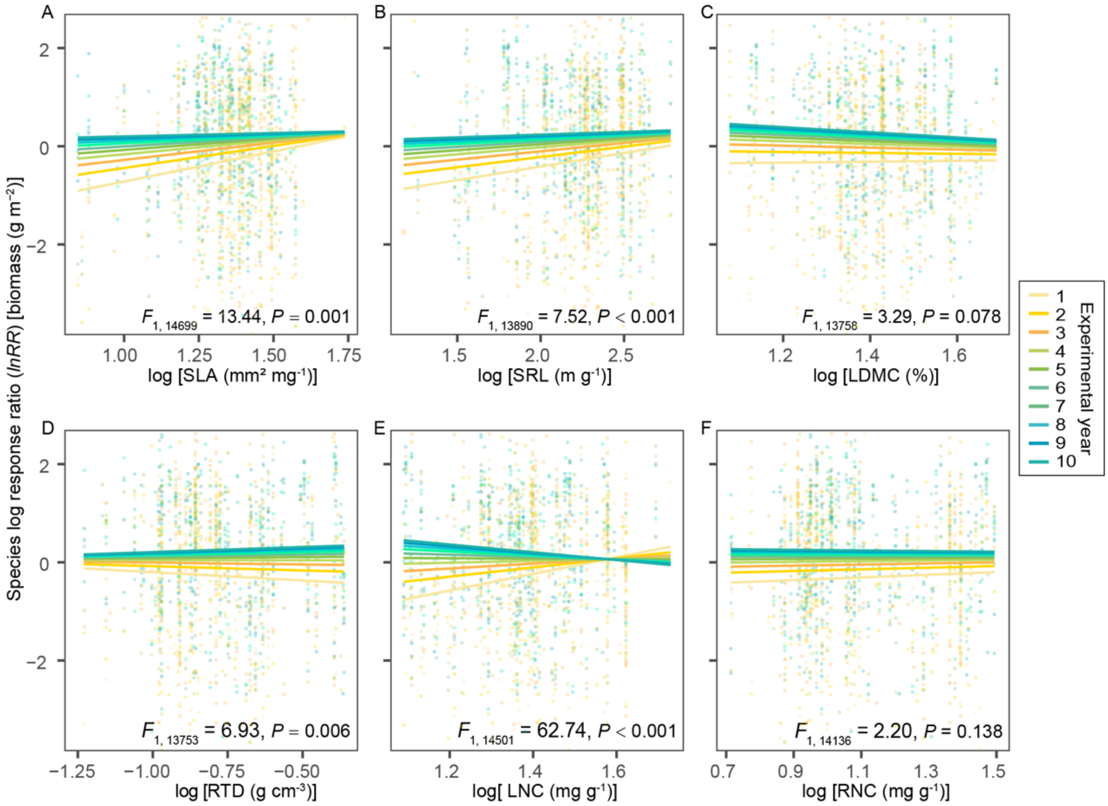

Supplementary Figure 12. Species overyielding (or underyielding) of aboveground biomass in relation to plant economics traits (only including measured values) across years in grassland experiments (n=39). Positive species log response ratio (*lnRR)* indicates overyielding and negative value indicates underyielding. Points represent the *lnRR* for each species and year in each experiment. *P* values are derived from the significance tests (one-sided *F*-test; *F* ratios with numerator degrees of freedom and denominator degrees of freedom are also shown) of the interaction term for plant economics traits and experimental years in the ANOVA test on linear mixed models. See the results with only long-term grassland experiments in supplementary figure 15.

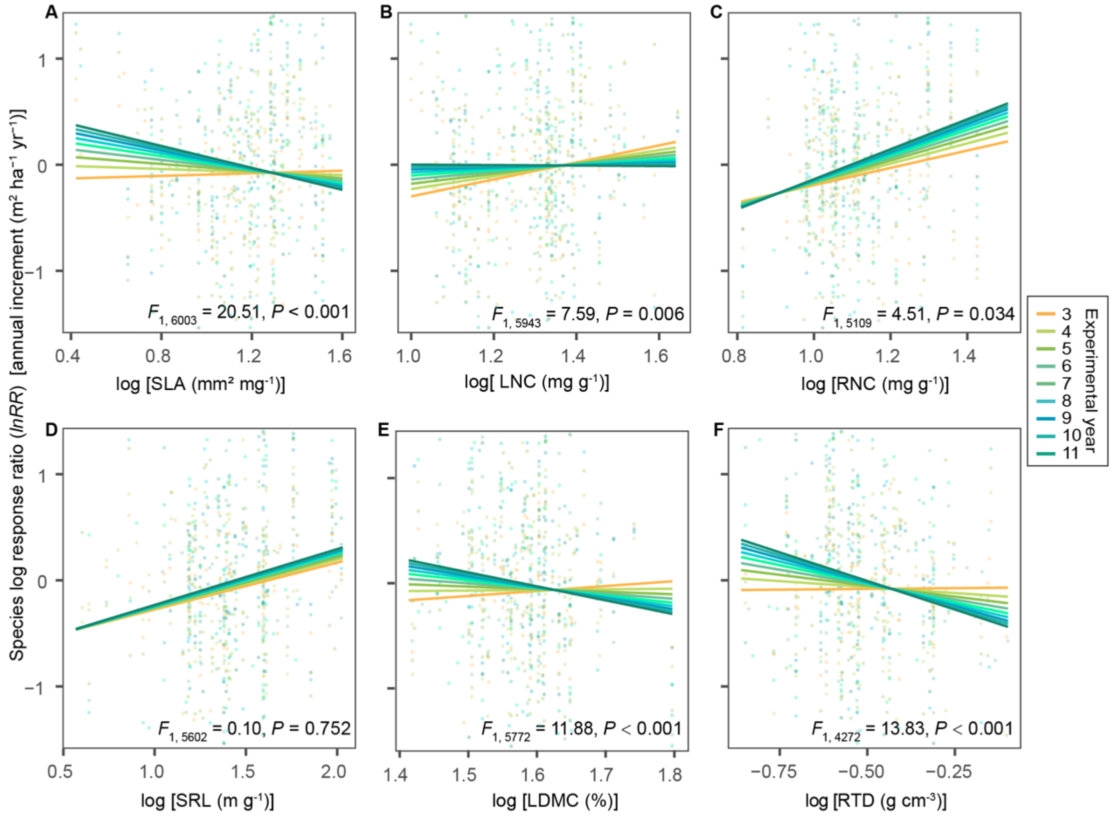

Supplementary Figure 13. Species overyielding (or underyielding) of annual increment of the basal area in relation to plant economics traits (only including measured values) across years in forest experiments (n=26). Positive species log response ratio (*lnRR)* indicates overyielding and negative value indicates underyielding. Points represent the *lnRR* for each species and year in each experiment. *P* values are derived from the significance tests (one-sided *F*-test; *F* ratios with numerator degrees of freedom and denominator degrees of freedom are also shown) of the interaction term for plant economics traits and experimental years in the ANOVA test on linear mixed models. Abbreviations of traits are given in Table S1.

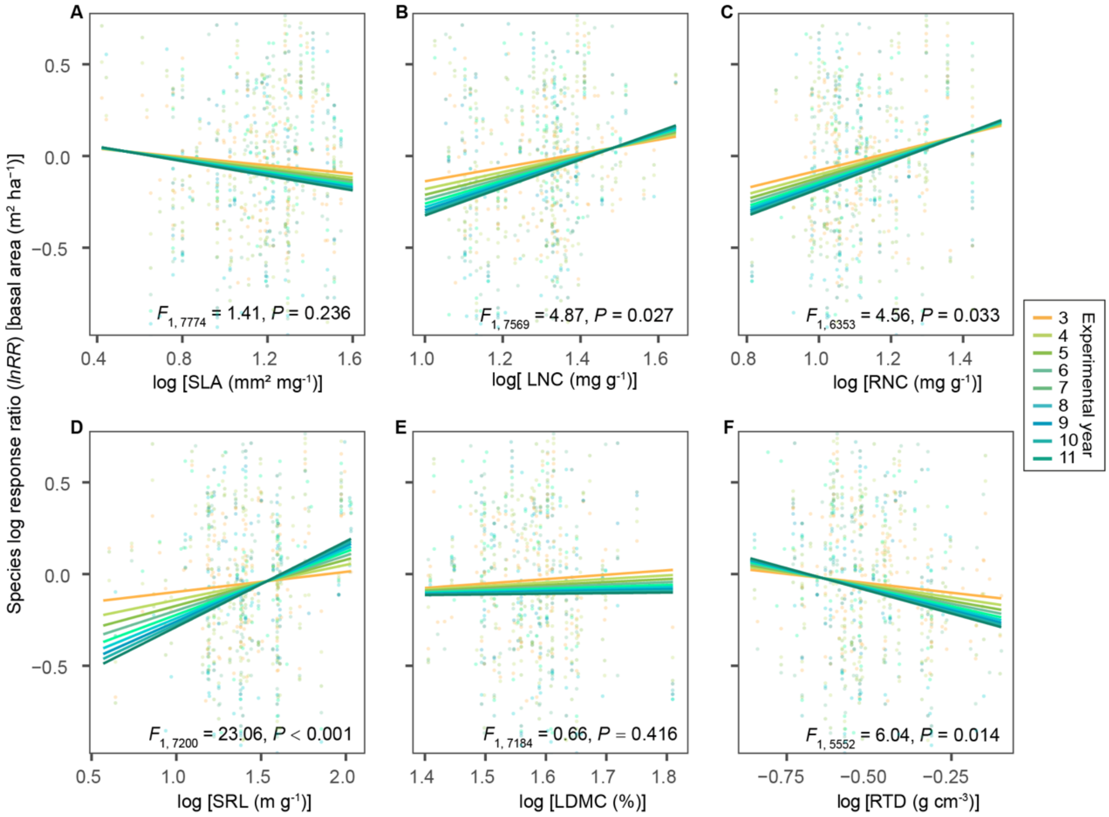

Supplementary Figure 14. Species overyielding (or underyielding) of accumulated total basal area in relation to plant economics traits (only including measured values) across experimental years in forests (n=26). Positive species log response ratio (*lnRR)* indicates overyielding and negative value indicates underyielding. Points represent the *lnRR* for each species and year in each experiment. *P* values are derived from the significance tests (one-sided *F*-test; *F* ratios with numerator degrees of freedom and denominator degrees of freedom are also shown) of the interaction term for plant economics traits and experimental years in the ANOVA test on linear mixed models.

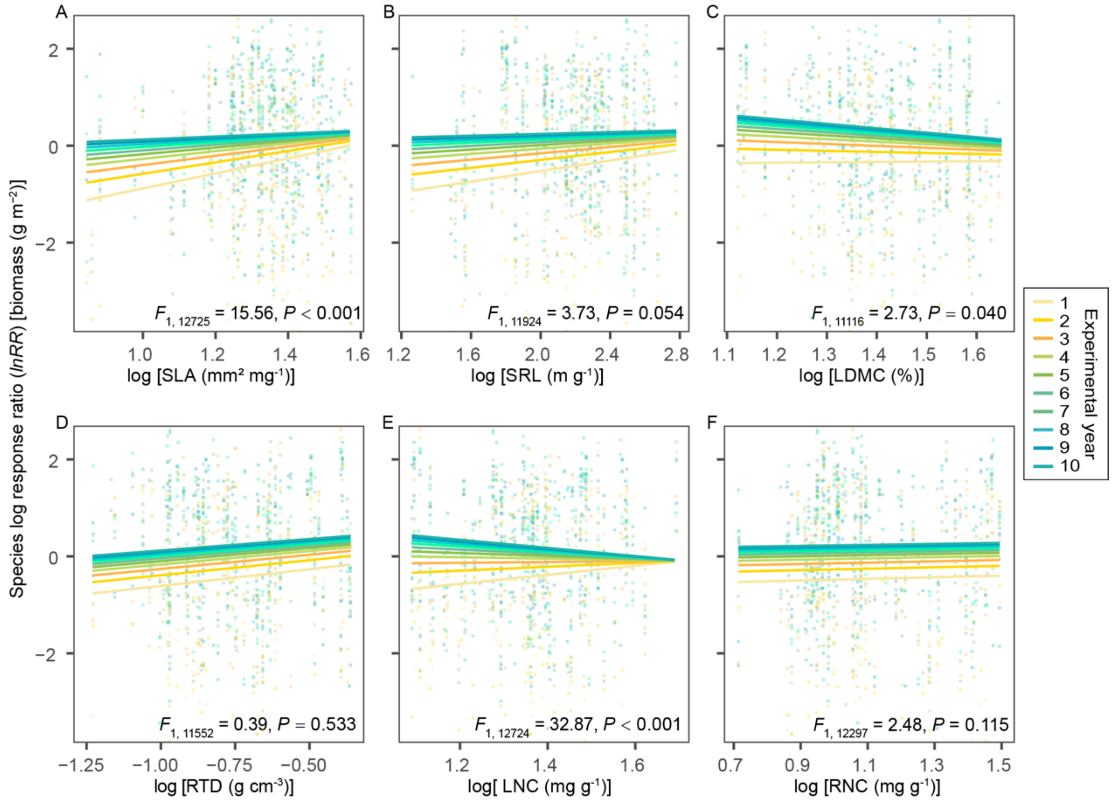

Supplementary Figure 15. Species overyielding (or underyielding) of aboveground biomass in relation to plant economics traits (only including measured values) across years in six longest grassland experiments (n=6). Positive species log response ratio (*lnRR)* indicates overyielding and negative value indicates underyielding. Points represent the *lnRR* for each species and year in each experiment. *P* values are derived from the significance tests (one-sided *F*-test; *F* ratios with numerator degrees of freedom and denominator degrees of freedom are also shown) of the interaction term for plant economics traits and experimental years in the ANOVA test on linear mixed models. See the results with the data from all grassland eperiments in supplementary figure 12.

Supplementary Methods

1. Reliability of the imputation of missing trait data

We assessed the reliability of the imputation of missing trait data using normalized root mean squared error. Following Carmona et al.[26], we artificially removed trait values from the trait dataset with complete empirical trait values and compared the difference between the artificially gap-filled data and the data with complete trait values. Firstly, we randomly selected the same number of random species from the incomplete data as from the complete data. Secondly, we artificially removed trait values following the patterns of missing trait values of the subset of species with incomplete information. Thirdly, we performed the imputation procedure using the missForest[28] approach in the artificial dataset. Finally, we compared the difference between real completed trait values and trait values with artificial removal and imputation. We used the normalized root mean square error (NRMSE) to estimate the average distance between real and artificially imputed values of species as a proportion of the range of trait values of species. We estimated the overall performance of the imputation procedure by repeating this procedure 1000 times and calculating a mean value of NRMSE of each trait, respectively. The results showed that the trait imputation for our trait dataset using missForest is acceptable. On average, the NRMSE for SLA, LNC, LDMC, SRL, RNC, and RTD of tree species was 0.01, 0.04, 0.05, 0.07, 0.08, and 0.08, respectively. The NRMSE for SLA, LNC, LDMC, SRL, RNC, and RTD of tree species was 0.01, 0.06, 0.09, 0.12, 0.13, and 0.14, respectively.

2. Estimation of accumulated basal area and annual basal area increment for tree diversity experiments

Basal area (BA; m^2 ha^{-1}) of each species on each plot was calculated using equation[29]

(1):

$$BA = 0.00007854 * \frac{\sum DBH^2}{a} \quad (1)$$

Where DBH is the diameter at breast height (cm) and a is the area of the plot (ha).

For experiments in which DBH is not available, we calculated basal area using basal diameter (cm). Although this probably overestimates basal area, it should not influence the temporal pattern of species- or community-level overyielding[29].

Annual basal area increment (IBA; m^2 ha^{-1} yr^{-1}) of each species on each plot was calculated using equation (2):

$$IBA = \frac{BA_2 - BA_1}{t_2 - t_1} \quad (2)$$

Where BA_2 and BA_1 are basal area at time 2 (t_2) and time 1 (t_1), respectively.

3. Quantification of complementarity and selection effects

We quantified complementarity (CE) and selection effects (SE) by using Loreau and Hector's additive partitioning method[30]:

$$CE = N\,\overline{\Delta RY}\,\overline{M} \quad (3)$$

$$SE = N\,cov(\Delta RY, M) \quad (4)$$

Where N is species richness, ΔRY is the difference between the observed and expected related yield of each species (calculated as $(P_{o,i}/M_i)-1/N$), and M is monoculture productivity.

4. Data analysis

We tested the effects of plant species richness on community-level productivity (as well as community overyielding) over time by using linear mixed-effect models. The model included plant species richness (SR), experimental year, and SR × year interaction as fixed factors. In addition, random terms were considered for each model, including a random intercept for each unique plot (i.e., PlotID), a random intercept for each site,

and random intercepts and slopes for SR and year per site (allows for differences between studies, which have different sown/plant densities and different climatic conditions within the site). We also accounted for the autocorrelation due to repeated measurements across years within each unique plotID by using a first-order autoregressive correlation structure with a time covariate in the residuals (i.e., Time). We started with a basic model which only included a random intercept for each unique plot, and then constructed additional models that progressively included a range of the other random terms and the temporal autocorrelation of residuals. Models with different random structures were compared using the Akaike information criterion (AIC). Models with the lowest AIC were selected as the final models, which include both random intercepts and slopes and the temporal autocorrelation of residuals.

We then used linear mixed-effect models to assess how species *lnRR* in mixtures were related to plant functional traits and how they change over time. The model included plant traits, experimental year, plant species richness (SR), traits × SR interaction, traits × year interaction, and SR × year interaction as fixed factors. SR was log2-transformed, plant traits were log10-transformed, and the experimental year was natural logarithm-transformed to meet the assumptions of linear mixed-effect models. In addition, random terms were considered for each model, including a random intercept for each species at each unique plot (i.e., SP_PlotID), a random intercept for each site, and random intercepts and slopes for SR, traits, and year per site. Furthermore, we accounted for the autocorrelation due to repeated measurements within each species of each unique plot across years by using a first-order autoregressive correlation with a time covariate in the residuals. We started with a basic model which only included a random intercept for each species in each unique plot, and then constructed additional models that progressively included a range of the other

random terms and the temporal autocorrelation of residuals. Models with the lowest AIC were selected as the final models, which include both random intercepts and slopes and the temporal autocorrelation of residuals.

R codes of linear mixed-effect models:

```
Model_community _grassland <- lme(LnRR _Biomass ~ SR*Year, random= list(~1|
        PlotID, ~ (1+SR+Year)|Site), correlation=corAR1(form = ~Time| PlotID),
        control =list(msMaxIter = 1000, msMaxEval = 1000),
        weights=varIdent(~Site), data=Grassland _community)

Model_community_forest_AI <- lme(LnRR_AI~ SR*Year, random= list(~1| PlotID,
        ~ (1+SR+Year)| Site), correlation=corAR1(form = ~Time| PlotID), control
        =list(msMaxIter = 1000, msMaxEval = 1000), weights=varIdent(~Site),
        data=Forest _community_AI)

Model_sp _community_ba <- lme(LnRR_ba~ SR*Year, random= list(~1| PlotID,~
        (1+SR+Year)| Site), correlation=corAR1(form = ~Time| PlotID), control
        =list(msMaxIter = 1000, msMaxEval = 1000), weights=varIdent(~Site),
        data=Forest_community_ba)

Model_sp _grassland <- lme(LnRR_Sp_Biomass ~ Trait*SR + Trait *Year +
        SR*Year, random = list(~1|SP_PlotID, ~(1+Trait+Year+SR)| Site),
        correlation=corAR1(form = ~Time|SP_ PlotID), control =list(msMaxIter =
        1000, msMaxEval = 1000), weights=varIdent(~Site), data=Grassland_species)

Model_sp _forest_AI <- lme(LnRR_ Sp_AI~ Trait*SR + Trait *Year+
        SR*Year, random= list(~1|SP_PlotID, ~ (1+Trait+Year+SR)| Site),
        correlation=corAR1(form = ~Time|SP_PlotID), control =list(msMaxIter =
        1000, msMaxEval = 1000), weights=varIdent(~Site),
        data=Forest_sp_species_AI)

Model_sp _forest_ba <- lme(LnRR_ Sp_ba~ Trait*SR + Trait *Year+
        SR*Year, random= list(~1|SP_PlotID, ~ (1+Trait+Year+SR)| Site),
        correlation=corAR1(form = ~Time|SP_ PlotID), control =list(msMaxIter =
        1000, msMaxEval = 1000), weights=varIdent(~Site),
        data=Forest_sp_species_ba)
```

References

1. Kirwan, L. *et al.* The Agrodiversity experiment: three years of data from a multisite study in intensively managed grasslands. *Ecology* **95**, 2680–2680 (2014).
2. Tilman, D. *et al.* The influence of functional diversity and composition on ecosystem processes. *Science* **277**, 1300–1302 (1997).
3. Reich, P. B. *et al.* Plant diversity enhances ecosystem responses to elevated CO2 and nitrogen deposition. *Nature* **410**, 809–810 (2001).
4. Hector, A. *et al.* Plant diversity and productivity experiments in European grasslands. *Science* **286**, 1123–1127 (1999).
5. Roscher, C. *et al.* The role of biodiversity for element cycling and trophic interactions: an experimental approach in a grassland community. *Basic Appl. Ecol.* **5**, 107–121 (2004).
6. Weisser, W. W. *et al.* Biodiversity effects on ecosystem functioning in a 15-year grassland experiment: Patterns, mechanisms, and open questions. *Basic Appl. Ecol.* **23**, 1–73 (2017).
7. Lanta, V. & Lepš, J. Effects of species and functional group richness on production in two fertility environments: an experiment with communities of perennial plants. *Acta Oecologica* **32**, 93–103 (2007).
8. Wilsey, B. J., Teaschner, T. B., Daneshgar, P. P., Isbell, F. I. & Polley, H. W. Biodiversity maintenance mechanisms differ between native and novel exotic-dominated communities. *Ecology Letters* **12**, 432–442 (2009).
9. Wilsey, B. J. & Polley, H. W. Reductions in grassland species evenness increase dicot seedling invasion and spittle bug infestation. *Ecology Letters* **5**, 676–684 (2002).

10. Jasper van Ruijven & Frank Berendse. Diversity-productivity relationships: Initial effects, long-term patterns, and underlying mechanisms. *Proc. Natl. Acad. Sci.* **102**, 695–700 (2005).
11. Devaney, J. L., Pullen, J., Cook-Patton, S. C., Burghardt, K. T. & Parker, J. D. Tree diversity promotes growth of late successional species despite increasing deer damage in a restored forest. *Ecology* **101**, e03063 (2020).
12. Scherer-Lorenzen, M., Schulze, E.-D., Don, A., Schumacher, J. & Weller, E. Exploring the functional significance of forest diversity: A new long-term experiment with temperate tree species (BIOTREE). *Perspect. Plant Ecol. Evol. Syst.* **9**, 53–70 (2007).
13. Forrester, D. I., Bauhus, J. & Khanna, P. K. Growth dynamics in a mixed-species plantation of Eucalyptus globulus and Acacia mearnsii. *For. Ecol. Manag.* **193**, 81–95 (2004).
14. Teuscher, M. *et al.* Experimental biodiversity enrichment in oil-palm-dominated landscapes in Indonesia. *Front. Plant Sci.* **7**, (2016).
15. Verheyen, K. *et al.* Assessment of the functional role of tree diversity: the multi-site FORBIO experiment. *Plant Ecol. Evol.* **146**, 26–35 (2013).
16. Binkley, D., Senock, R., Bird, S. & Cole, T. G. Twenty years of stand development in pure and mixed stands of Eucalyptus saligna and nitrogen-fixing Facaltaria moluccana. *For. Ecol. Manag.* **182**, 93–102 (2003).
17. Tobner, C. M., Paquette, A., Reich, P. B., Gravel, D. & Messier, C. Advancing biodiversity–ecosystem functioning science using high-density tree-based experiments over functional diversity gradients. *Oecologia* **174**, 609–621 (2014).
18. Epron, D. *et al.* Partitioning of net primary production in Eucalyptus and Acacia stands and in mixed-species plantations: Two case-studies in contrasting tropical

environments. *For. Ecol. Manag.* **301**, 102–111 (2013).

19. Hantsch, L. *et al.* Tree diversity and the role of non-host neighbour tree species in reducing fungal pathogen infestation. *J. Ecol.* **102**, 1673–1687 (2014).
20. Piotto, D., Craven, D., Montagnini, F. & Alice, F. Silvicultural and economic aspects of pure and mixed native tree species plantations on degraded pasturelands in humid Costa Rica. *New Forests* **39**, 369–385 (2010).
21. Ferlian, O. *et al.* Mycorrhiza in tree diversity–ecosystem function relationships: conceptual framework and experimental implementation. *Ecosphere* **9**, e02226 (2018).
22. Perring, M. P. *et al.* The Ridgefield Multiple Ecosystem Services Experiment: Can restoration of former agricultural land achieve multiple outcomes? *Agric. Ecosyst. Environ.* **163**, 14–27 (2012).
23. Healy, C., Gotelli, N. J. & Potvin, C. Partitioning the effects of biodiversity and environmental heterogeneity for productivity and mortality in a tropical tree plantation. *J. Ecol.* **96**, 903–913 (2008).
24. Vehviläinen, H. & Koricheva, J. Moose and vole browsing patterns in experimentally assembled pure and mixed forest stands. *Ecography* **29**, 497–506 (2006).
25. Parrotta, J. A. Productivity, nutrient cycling, and succession in single- and mixed-species plantations of Casuarina equisetifolia, Eucalyptus robusta, and Leucaena leucocephala in Puerto Rico. *For. Ecol. Manag.* **124**, 45–77 (1999).
26. Carmona, C. P. *et al.* Fine-root traits in the global spectrum of plant form and function. *Nature* **597**, 683–687 (2021).
27. Stekhoven, D. J. & Bühlmann, P. MissForest—non-parametric missing value imputation for mixed-type data. *Bioinformatics* **28**, 112–118 (2012).

28. Bettinger, P., Boston, K., Siry, J. P. & Grebner, D. L. Chapter 2 - Valuing and Characterizing Forest Conditions. in *Forest Management and Planning (Second Edition)* (eds. Bettinger, P., Boston, K., Siry, J. P. & Grebner, D. L.) 21–63 (Academic Press, 2017).
29. Guerrero-Ramirez, N. R. *et al.* Diversity-dependent temporal divergence of ecosystem functioning in experimental ecosystems. *Nat. Ecol. Evol.* **1**, 1639–1642 (2017).
30. Loreau, M. & Hector, A. Partitioning selection and complementarity in biodiversity experiments. *Nature* **412**, 72–76 (2001).
