# Supplementary material for: Effects of plant diversity on productivity strengthen over time due to trait-dependent shifts in species overyielding
Source: Nat Commun. 2024 Mar 7;15:2078. doi: 10.1038/s41467-024-46355-z (PMC10920907; doi:10.1038/s41467-024-46355-z)
Supplement: Supplementary file 3 — Reporting Summary [file 41467_2024_46355_MOESM3_ESM.pdf]

nature portfolio

Corresponding author(s): Liting Zheng, Enrong Yan

Last updated by author(s): Feb 6, 2024

Reporting Summary

Nature Portfolio wishes to improve the reproducibility of the work that we publish. This form provides structure for consistency and transparency in reporting. For further information on Nature Portfolio policies, see our Editorial Policies and the Editorial Policy Checklist.

Statistics

For all statistical analyses, confirm that the following items are present in the figure legend, table legend, main text, or Methods section.

n/a	Confirmed	
☐	☒	The exact sample size (*n*) for each experimental group/condition, given as a discrete number and unit of measurement
☐	☒	A statement on whether measurements were taken from distinct samples or whether the same sample was measured repeatedly
☐	☒	The statistical test(s) used AND whether they are one- or two-sided<br>*Only common tests should be described solely by name; describe more complex techniques in the Methods section.*
☐	☒	A description of all covariates tested
☐	☒	A description of any assumptions or corrections, such as tests of normality and adjustment for multiple comparisons
☐	☒	A full description of the statistical parameters including central tendency (e.g. means) or other basic estimates (e.g. regression coefficient) AND variation (e.g. standard deviation) or associated estimates of uncertainty (e.g. confidence intervals)
☐	☒	For null hypothesis testing, the test statistic (e.g. *F*, *t*, *r*) with confidence intervals, effect sizes, degrees of freedom and *P* value noted<br>*Give P values as exact values whenever suitable.*
☒	☐	For Bayesian analysis, information on the choice of priors and Markov chain Monte Carlo settings
☐	☒	For hierarchical and complex designs, identification of the appropriate level for tests and full reporting of outcomes
☐	☒	Estimates of effect sizes (e.g. Cohen's *d*, Pearson's *r*), indicating how they were calculated

Our web collection on statistics for biologists contains articles on many of the points above.

Software and code

Policy information about availability of computer code

Data collection	No software was used for data collection.
Data analysis	The data analysis was conducted in R 4.2.1 (R Core Team, 2022), including 'missForest','V.Phylomaker','nlme' packages. Details are reported in the Methods section. R code of the linear mixed-effects models is provided in the Supplementary Methods.

For manuscripts utilizing custom algorithms or software that are central to the research but not yet described in published literature, software must be made available to editors and reviewers. We strongly encourage code deposition in a community repository (e.g. GitHub). See the Nature Portfolio guidelines for submitting code & software for further information.

Data

Policy information about availability of data

All manuscripts must include a data availability statement. This statement should provide the following information, where applicable:

- Accession codes, unique identifiers, or web links for publicly available datasets
- A description of any restrictions on data availability
- For clinical datasets or third party data, please ensure that the statement adheres to our policy

The information for each experiment, interannual climate data, and species-level trait data used in this study are archived in Figshare (https://doi.org/10.6084/m9.figshare.25148690). The public trait data can be obtained from the TRY Plant Trait Database (https://www.try-db.org/TryWeb/), the Global Root Trait (GRooT) database (https://groot-database.github.io/GRooT/), the Botanical Information and Ecology (BIEN) Network (https://bien.nceas.ucsb.edu/bien/), and AusTraits

database (https://austraits.org/). Unpublished productivity data of grassland and tree biodiversity experiments will be available from the corresponding author upon request. The unpublished productivity data of the tree diversity experiments within the TreeDivNet could be reached by contacting the network coordinators (https://treedivnet.ugent.be/contact).

Research involving human participants, their data, or biological material

Policy information about studies with human participants or human data. See also policy information about sex, gender (identity/presentation), and sexual orientation and race, ethnicity and racism.

Reporting on sex and gender	N/A
Reporting on race, ethnicity, or other socially relevant groupings	N/A
Population characteristics	N/A
Recruitment	N/A
Ethics oversight	N/A

Note that full information on the approval of the study protocol must also be provided in the manuscript.

Field-specific reporting

Please select the one below that is the best fit for your research. If you are not sure, read the appropriate sections before making your selection.

☐ Life sciences ☐ Behavioural & social sciences ☒ Ecological, evolutionary & environmental sciences

For a reference copy of the document with all sections, see nature.com/documents/nr-reporting-summary-flat.pdf

Ecological, evolutionary & environmental sciences study design

All studies must disclose on these points even when the disclosure is negative.

Study description	Using temporal data from 65 long-term grassland and forest experimental ecosystems, this study shows that temporal changes in species overyielding are responsible for the observed increases in productivity in diverse plant communities over time. Furthermore, differences in temporal trends of species overyielding are shaped by plant ecological strategies, captured using leaf and root economic traits.<br>We assembled a database by combining data from both grassland and forest biodiversity experimental ecosystems. Experiments included in this study met the following criteria: (1) plant species richness was directly manipulated through sowing or planting and contained both mixtures and corresponding monocultures, (2) the relative abundance of species in each mixture was known, (3) aboveground plant biomass for both species- and plot-level were available, and (4) the experiment was conducted for at least three years with measurements performed in at least two different years. These experiments typically prevent plot contamination by repeatedly removing non-target species to maintain the desired composition and diversity levels. When an experiment included other treatments (e.g., fertilization), we filtered out data where other treatments existed. For grassland experiments, we used up to ten years of data per study with a range in species richness from one to 16 (if available) to reduce potential bias associated with the scarcity of experiments that lasted longer than ten years. Similarly, for forest experiments, we used up to eleven years of data with a range in species richness from one to eight (if available) and excluded data from the initial two experimental years. In total, we used data from 39 grassland and 26 forest experiments for the main analysis (see the details of the experiments included in this study in Supplementary Table 1).
Research sample	We assembled a database by combining original data on the aboveground productivity of each species in every plot from all experiments. For grasslands, the principle investigator at each site measured annual peak aboveground live biomass (g m-2) as a measure of species-level productivity within each plot of each year. The summed biomass for all species per plot each year was determined as grassland community-level productivity for each year. In forests, the principle investigator at each site measured annual basal area increment and the accumulated basal area as estimates of species-level productivity within each plot of each year (Supplementary Methods 2).
Sampling strategy	The experiments included in this study typically prevent immigration by repeatedly removing non-target species to maintain the composition. Species-level trait values were obtained from TRY, GRooT, the Botanical Information and Ecology Network (BIEN), and AusTraits databases, or site-specific measurements when available.
Data collection	In total, we used data from 39 grassland and 26 forest experiments. Data collection was done by the principle investigator at each site. For grassland communities, the annual peak aboveground live biomass of each species were measured as a measure of species-level productivity within each plot for each year. In forests, the basal diameter of each individual with each plot was measured and the annual basal area increment and the total accumulated basal area were calculated as estimates of species-level productivity within each plot of each year (see details in Supplementary Methods 2).

Timing and spatial scale	For grassland experiments, the harvest to measure annual peak aboveground live biomass is usually done in 1 m2 subplots. For forest experiments, the measurement of basal diameter is done at the individual level within each plot. The plot size is different among experiments, ranging from 16 m2 to 2025 m2. Plot size of each experiment within the TreeDivNet can be obtained from the website(https://treedivnet.ugent.be/). And the time scale of the data of each experiment change from three years to 18 years (see the details of the experiments included in this study in Supplementary Table 1). The whole dataset using in this study was assembled from April 2021 to September 2023.
Data exclusions	Exclusion criteria were pre-established. Experiments included in this study met the following criteria: (1) plant species richness was directly manipulated through sowing or planting and contained both mixtures and corresponding monocultures, (2) the relative abundance of species in each mixture was known, (3) aboveground plant biomass for both species- and plot-level were available, and (4) the experiment was conducted for at least three years with measurements performed in at least two different years. We excluded the first two years to avoid potential biases associated with re-planting that commonly occurred in the initial two years of some forest experiments. Data exclusions for the data analysis: Species lnRR approaches infinity when monoculture productivity is near zero or negative infinity when the productivity of a species in the mixture is near zero (establishment failure). Consequently, we excluded data points that were above the 99.5% percentile and lower than the 0.5% percentile.
Reproducibility	All attempts to repeat the analysis and results were successful. The R code to conduct the linear mixed effect model were shown in the Supplementary Method.
Randomization	We tested the hypotheses in this study by using temporal data from 65 biodiversity experimental ecosystems across the globe that manipulated plant species richness in grassland and forest communities. The data analysis were conducted with random effect models (R code to conduct the linear mixed effect model were shown in the Supplementary Methods 4).
Blinding	To fill the gaps in the trait datasets for analysis. We excluded species for which trait values could not be reliably imputed, because they did not have any trait measurements. We then assessed the reliability of the imputation procedure (see details in Supplementary Methods 1).

Did the study involve field work? ☒ Yes ☐ No

Field work, collection and transport

Field conditions	This study used temporal data from 65 biodiversity experiments across the globe that manipulated plant species richness in grassland (39) and forest (26). The experimental sites cover a wide range of climatic conditions and locations. The information for each experiment are archived in Figshare (https://doi.org/10.6084/m9.figshare.25148690).
Location	The location of each experiment are archived in Figshare (https://doi.org/10.6084/m9.figshare.25148690).
Access & import/export	Access to site and data collection by the principle investigator at each site followed standard practices and complies with laws.
Disturbance	There was no disturbance during the experimental period. Walkways were established to minimize sampling effects from the data collection.

Reporting for specific materials, systems and methods

We require information from authors about some types of materials, experimental systems and methods used in many studies. Here, indicate whether each material, system or method listed is relevant to your study. If you are not sure if a list item applies to your research, read the appropriate section before selecting a response.

Materials & experimental systems

n/a	Involved in the study	
☒	☐	Antibodies
☒	☐	Eukaryotic cell lines
☒	☐	Palaeontology and archaeology
☒	☐	Animals and other organisms
☒	☐	Clinical data
☒	☐	Dual use research of concern
☐	☒	Plants

Methods

n/a	Involved in the study	
☒	☐	ChIP-seq
☒	☐	Flow cytometry
☒	☐	MRI-based neuroimaging

Dual use research of concern

Policy information about dual use research of concern

Hazards

Could the accidental, deliberate or reckless misuse of agents or technologies generated in the work, or the application of information presented in the manuscript, pose a threat to:

No	Yes	
☒	☐	Public health
☒	☐	National security
☒	☐	Crops and/or livestock
☒	☐	Ecosystems
☒	☐	Any other significant area

Experiments of concern

Does the work involve any of these experiments of concern:

No	Yes	
☒	☐	Demonstrate how to render a vaccine ineffective
☒	☐	Confer resistance to therapeutically useful antibiotics or antiviral agents
☒	☐	Enhance the virulence of a pathogen or render a nonpathogen virulent
☒	☐	Increase transmissibility of a pathogen
☒	☐	Alter the host range of a pathogen
☒	☐	Enable evasion of diagnostic/detection modalities
☒	☐	Enable the weaponization of a biological agent or toxin
☒	☐	Any other potentially harmful combination of experiments and agents
